# Relation between BCG vaccine scar and an interferon-gamma release assay in immigrant children with “positive” tuberculin skin test (≥10 mm)

**DOI:** 10.1186/s12879-016-1872-9

**Published:** 2016-10-06

**Authors:** Margret Johansson Gudjónsdóttir, Karsten Kötz, Ruth Stangebye Nielsen, Philip Wilmar, Sofia Olausson, Daniel Wallmyr, Birger Trollfors

**Affiliations:** 1 Department of Paediatrics, Institute of Clinical Sciences, Sahlgrenska Academy, Gothenburg University, Gothenburg, Sweden; 2 Department of Paediatrics, Angered Hospital, Gothenburg, Sweden; 3 Department of Child Health, South Älvsborg Hospital, Borås, Sweden; 4 Department of Pediatrics, Neonatology, Sahlgrenska University Hospital, 41685 Gothenburg, Sweden

**Keywords:** Tuberculosis, BCG scar, Tuberculin skin test, Quantiferon

## Abstract

**Background:**

Immigrants from countries with high incidence of tuberculosis (TB) are usually offered screening when they arrive to low incidence countries. The tuberculin skin test (TST) is often used. The interferon gamma release assays (IGRAs) are more specific and not affected by BCG vaccination. The aims of this study were 1. To see if there if there is a correlation between a positive IGRA (QFT) and presence of a BCG scar in children with TST ≥10 mm, 2. To compare the TST diameter with QFT result, 3. To see if chest X-ray can be omitted in QFT negative children despite TST ≥10 mm.

**Methods:**

762 healthy children/adolescents (median age 14 years) arriving to Gothenburg and surroundings with TST ≥10 mm were tested with QFT.

**Results:**

A total of 163/492 (33 %) children with BCG scar had positive QFT, whereas 205/270 (76 %) without BCG scar had positive QFT (*p <* 0.0001). The median TST was 12 mm in QFT negative and 18 mm in QFT positive children (*p <* 0.0001) but with considerable overlap. Median TST was the same (12 mm) in QFT negative children with and without BCG scar. Among the QFT positive children 25/368 had chest X-ray changes compared to 2/393 among the QFT negative children (*p <* 0.0007).

**Conclusions:**

Previous BCG vaccination had an effect on the TST diameter so an IGRA is recommended to diagnose latent TB. Using only TST for screening of latent TB would lead to overdiagnosis. The TST diameter was larger in QFT positive than in QFT negative children but could not predict QFT in the individual patient. Chest X ray contributes little to the diagnosis of TB in QFT negative children but can not be omitted because of late seroconversion of QFT in some patients.

**Trial registration:**

Not applicable.

## Background

Immigrants from countries with high incidence of tuberculosis (TB) are offered medical examination and screening for TB and other infectious diseases when they arrive to Sweden. The screening for TB consisted until recently of physical examination and a tuberculin skin test (TST). If TST was ≥ 10 mm a chest X ray was performed. If there were no signs of active TB, preventive chemotherapy was recommended to children and adolescents (in the following called children) if compliance could be expected to be good. From 2007 children coming to the Gothenburg area with TST ≥10 mm, irrespective if a BCG scar was visible or not, were referred to clinics specialising in TB where Quantiferon was assayed. Preventive chemotherapy was offered if Quantiferon was positive. The TST limit of 10 mm follows the recommendations of the National Board of Health and Welfare.

TST is not ideal for screening for latent TB. It requires two visits and it crossreacts with antigens in the Bacillus Calmette-Guérin (BCG) vaccine and with many nontuberculous mycobacteria (NTM) [1–8]. Repeated testing can induce false positive results. Furthermore, the definition of a positive test varies from 5 to 15 mm) [1–8]. The US Centers for Disease Control and Prevention adjust for underlying diseases, history of vaccination and origin of the immigrant (http://www.cdc.gov/tb/publications/factsheets/testing/skintesting.htm). In the UK, 5 mm is used as cut-off in children with BCG scar (National Institure for Health and Care Excellence, https://www.nice.org.uk/guidance/ng33/chapter/recommendations#diagnosing-latent-tb-in-children-and-young-people).

The interferon gamma release assays (IGRAs) solve many, but not all, problems in the evaluation of latent TB. They require only one visit, do not cross-react with BCG vaccines and cross-react with fewer NTM than TST. Several studies have shown a rather good correlation between TST and IGRAs except in very young children [9–13] but a recent study in 126 adults showed a relatively poor correlation between TST and IGRAs and also between two IGRAs (QuantiFERON -TB Gold In-Tube® (QFT) and T-SPOT.TB®) [12]. The use of IGRAs is therefore controversial and has not replaced TST for diagnosis of latent TB in many countries [14, 15].

It is usually difficult to obtain a reliable vaccination history from the accompanying adult family member and impossible if the child arrives alone to Sweden. The only way to obtain information about BCG vaccination is to look for typical BCG scars even though BCG vaccination does not always result in a scar and scars can wane with time [1, 16–18].

## Methods

### The aim of the study

The three questions in the present study were; 1. Is there a correlation between presence of a BCG scar and positive QFT? (The presence of a BCG scar was in the absence of documented BCG vaccination used as a substitute for vaccination), 2. Does the size of the TST differ between children with positive and negative QFT? 3. Can chest X ray be omitted in children with negative QFT, as has been suggested for adults [12]?

### Study design and setting

The study is a retrospective evaluation of patient records of immigrant children who had undergone health examination and been found to have TST ≥10 mm. The initial health examination including TST of immigrant children in the area is performed at several Primary Health Clinics. Children with TST ≥10 mm are referred to the Departments of Pediatrics of Sahlgrenska University Hospital or the outpatient clinics in Angered (north-east Gothenburg), Borås or Alingsås (all serving surrounding communities). A chest X-ray is performed. The study included children examined from December 2007 through May 2016. The inclusion criteria in the study were 1) TST ≥10 mm, 2) No clinical symptoms (cough, swollen lymph nodes, loss of weight and appetite, fatigue, fever) or findings on physical examination compatible with active tuberculosis, 3) No known exposure to tuberculosis before or after arrival to Sweden. Three children, who had come to Sweden alone, who told that they had been treated for many months in their home countries with medicines which made their urine red (assumed to be Rifampicin), were not included. One child was excluded because it had several scars after recent varicella. It was not possible to evaluate if any of these scars were caused by BCG. There are no reliable statistics about the number of immigrant children who undergo health examinations in the area but an estimate indicates that it is about 600 children (70 % of all immigrant children) per year increasing the last years (personal communication: Leif Dotevall, MD, Department of Communicable Diseases, Västra Götaland, Sweden).

### Patients

A total of 762 children were included in the study. Gender and age distribution is shown in Table 1. As many as 215 children came alone without an accompanying adult (Table 1). Most of the unaccompanied minors came from Somalia (121), Afghanistan (48) and Eritrea (16). These children were all aged between 11 and 18 years.

**Table 1:** Base-line characteristics of the 762 children included in the study

Boys-girls	458 boys, 304 girls
Median age	14 years
10–18 years	625
5–9 years	99
0–4 years	38
Median time^a^ spent in Sweden	8 (0–96) months
Unaccompanied minors	215 (46 girls, 169 boys)
Concomitant diseases	36
HBsAg positive	8
Asthma	3
Rickets	3
Kidney stones	4
Epilepsia	3
Iron deficiency anema	3
Thalassemia minor	2
Mb Down	2
Coeliaci	1
Psychomotor retardation	2
Duchenne’s muscular dystrophy	1
Diabetes mellitus	1
Cannabis smoker	1
Mb Crohn	1
Cerebral palsy	1

^a^Time (range) when QFT was assayed

Chest X ray was performed in all but one child, whose mother declined X ray. This child had a scar and was QFT negative.

Both arms were inspected for scars compatible with BCG vaccination. If no scars were found on the arms, the thighs were inspected except in a few teen-age girls who refused this. They all said that they had no scars on the legs, which was accepted as absence of scar. The evaluation of BCG scar was made before the QFT result was known. Only four children had written documentation of BCG vaccination in their home countries. One of them had no scar and is included among the children without scar. The other three had a scar. Three children had scars on the dorsal side of the lower arm and one on the hip. They were all accompanied by their mothers who knew that these scars were induced by vaccination against TB.

The median time in Sweden was 8 (range 0–96) months when the QFT was performed. All patients were tested for syphilis, hepatitis B and HIV. No patient was syphilis or HIV infected. There were 36 children with chronic diseases (Table 1). The eight HBsAg positive patients were all asymptomatic and had normal liver function tests.

The children came from 59 different countries, when political borders and not ethnicity was considered. Chechnya and Dagestan was included in Russia and Kosovo in Serbia. Kurds and Romanis were included in the countries they came from. The only exception was 23 children from the Ogaden region, Ethiopia, who were included among Somalians. The largest groups came from Somalia + Ogaden (349), Afghanistan (77), Iraq (34) and Eritrea (24). Table 2 shows numbers of children from different regions of the world and the presence of BCG scars.

**Table 2:** Frequency of BCG scars

	Number with scar/total number from this region	Proportion with scar
Somalia	193/349	55 %
Middle East + North Africa	140/190	74 %
Rest of Africa	57/99	58 %
South East Asia	40/49	82 %
Europe	41/50	82 %
China + Taiwan + Mongolia	9/11	82 %
India + Nepal	5/7	71 %
South America	7/7	100 %

Numbers and proportions of children with BCG scar from different parts of the world

### Tuberculin skin test

0.1 ml containing 2 TU PPD RT 23 (Statens Seruminstitut, Copenhagen, Denmark) was injected intracutaneously into the dorsal side of the lower arm. The diameter of the swelling was measured after 72 h. Towards the end of the study there was a lack of the TST reagents from Statens Seruminstitut. During that time Tubertest from Sanofi, Lyon, France was used.

### Quantiferon

Blood for QFT was obtained ≥ 2 months after TST was read. The sampling and analyses were performed with QuantiFERON®-TB Gold In Tube according to the instructions of the manufacturer (Cellestis Ltd., Chadstone, Victoria, Australia). One mL blood was collected into each of three different collection tubes, which include Nil Control tube, TB Antigen tube, and Mitogen (positive control) tube. The samples were transported to the laboratory within 16 h. At the laboratory, the collection tubes were incubated at 37° for 16–20 h and subsequently centrifuged at 2300 g for 15 min. The concentration of interferon gamma in the resulting plasma in the tubes was measured by ELISA and related to a concentration standard. The absorbance was measured in Vmax Kinetic Microplate Reader (Molecular Devices, USA) and the optical density (OD) values were evaluated using the QFT®-TB Gold Analysis Software version 2.50. According to the producer, QFT is positive when TB-antigen – Nil is ≥ 0.35 U/mL. In this study new blood samples were obtained and the test was repeated when TB-antigen – Nil was 0.35 to ≤ 1.0 U/mL. This was seen in 18 patients. Seventeen of them were clearly negative in the second test and one was clearly positive. The results of the second test were used in the study.

### Statistics

Proportions were compared with 2-tailed Fisher’s exact test using the method of summing small P values and median values with 2-tailed Mann–Whitney U test.

## Results

A total of 762 children fulfilled all inclusion criteria. BCG scars were found in 492 of them (65 %) (Table 2). Scars were found in significantly lower proportions in children from Somalia and the rest of Sub-Saharan Africa (56 %) than in all other children combined (77 %) (*p <* 0.0001).

QFT was positive in 163/492 (33 %) children with a BCG scar and in 205/270 (76 %) without a scar (*p <* 0.0001). Significant differences were found in children from Somalia, the Middle East + North Africa, Sub-Saharan Africa, Southeast Asia, Europe and China. Children from India and South America were too few for separate calculations (Table 3). Somalian children with a BCG scar were significantly more often QFT positive (55 %) than all other groups combined (22 %) (*p <* 0.0001). Significant differences of the same magnitude were found in boys and girls.

**Table 3:** Relation between BCG scar and outcome of Quantiferon

	With scar	Without scar	
	Pos QFT/total number	Pos QFT/total number	*p*-value
Somalia	97/193 (50 %)	129/156 (83 %)	<0.0001
Middle East + North Africa	34/140 (24 %)	34/50 (68 %)	<0.0001
Rest of Africa	17/57 (30 %)	27/42 (64 %)	0.001
South East Asia	10/40 (25 %)	8/9 (89 %)	0.0007
Europe	4/41 (10 %)	4/9 (44 %)	0.0264
China + Taiwan + Mongolia	0/9 (0 %)	2/2 (100 %)	0.0182
India + Nepal	0/5 (0 %)	1/2 (50 %)	ns
South America	1/7 (14 %)	0	ns
All boys	90/291 (31 %)	127/167 (76 %)	<0.0001
All girls	73/201 (36 %)	78/103 (76 %)	<0.0001
ALL CHILDREN	163/492 (33 %)	205/270 (76 %)	<0.0001

Positive Quantiferon (number out of total (%) with positive QFT in children with and without scar arriving to Sweden from different parts of the world. All children had TST ≥10 mm. (p-values refer to comparisons of proportions in children with and without BCG scar in the respective population)

Among children with a scar and TST ≥ 10 mm 329/492 (67 % had negative QFT, while only 65/270 (24 %) of the children without a scar and TST ≥ 10 mm had negative QFT (*p <* 0.0001).

Among children 5 – 9 years 19/58 (33 %) with BCG scar had positive QFT compared to 28/41 (68 %) children without scar (*p =* 0.0006). A statistical comparison for children < 5 years of age was not meaningful because 34/36 with scar had negative QFT while one of two without scar had negative QFT.

Among the 215 unaccompanied children aged 11 – 18 years 131/215 (61 %) had positive Quantiferon compared to 179/383 (47 %) children aged 11–18 years who came with their families (*p =* 0.0009). Among those who came with their families and were aged between 11 and 18 years, the proportion with BCG scars was higher 266/383 (69 %) than in the unaccompanied children, 113/215 (53 %) (*p <* 0.0001).

**Fig. 1:**
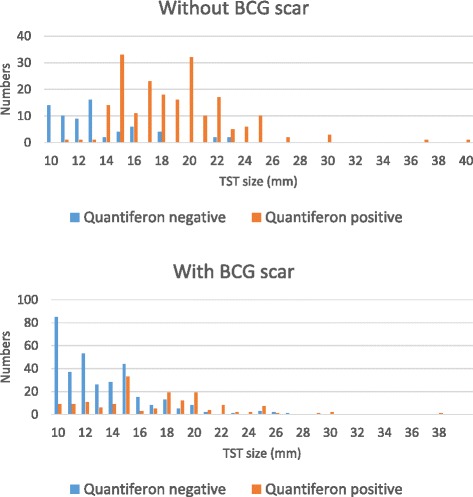
TST diameters in Quantiferon negative and positive children

The median TST diameters were significantly higher in QFT positive than in QFT negative children (*p <*0.0001) both in children with and without scar (Table 4). There was, however, a considerable overlap (Fig. 1). Among the children with negative QFT, 279 had TST 10–14 mm and 115 had TST ≥ 15 mm.

**Table 4:** TST diameter related to BCG scar and Quantiferon

	QFT Negative	QFT Positive	*p* - value
With BCG scar	12 (10–27)	17 (10–38)	<0.0001
Without BCG scar	12 (10–23)	18 (10–40)	<0.0001

Relation between median (range) TST diameter (mm) and QFT result in children with and without BCG scar and positive and negative Quantiferon

Chest X ray changes were seen in 25/368 QFT positive and in 2/394 QFT negative children. The changes consisted of enlarged hilar or tracheal lymph nodes or mild to moderate inflammatory infiltrates. One QFT negative child (6 year old boy from Somalia) had a TST just at the cut-off value of 10 mm. No mycobacteria could be isolated from bronchoalveolar lavage. He had elevated erythrocyte sedimentation rate (ESR) and C- Reactive Protein Test (CRP). His changes disappeared after 6 months of antituberculosis treatment for active TB with initially four drugs. This QFT negative child became QFT positive after 1.5 years, when he had finished treatment for active TB. The other QFT negative child (15 years old boy from Somalia) had a small basal inflammatory infiltrate. He had both ESR < 15 mm/h and CRP < 1.0 mg/L on repeated testing. He became QFT positive after 7 weeks. No mycobacteria were isolated from bronchoalveolar lavage. He was also treated for active TB with 4 drugs initially and chest X ray normalised.

Bronchoalveolar lavage was performed in 22 of the 25 QFT positive children with X ray changes. *Mycobacterium tuberculosis* was cultured from one of them. He was treated as a case of active TB and chest X-ray normalised. The 21 culture-negative children all had normal ESR and CRP. All of them were treated as active TB and all had normal chest X ray after 6 months.

## Discussion

The most important difficulty in comparing TST and QFT is due to the absence of a “golden standard” for the diagnosis of latent TB. Based on theoretical assumptions a positive QFT is, however, more specific than a large TST which has no generally approved definition of “positive” and no positive and negative controls. This must be kept in mind when the present and a very large number of comparative studies are evaluated.

The study showed that there is a highly significant correlation between presence of BCG scar and positive QFT. The reason for this could not be answered by the results. The finding that a higher proportion of children with scar had negative QFT than children without scar (67 % verus 24 %) speaks in favour of the possibility that BCG vaccination resulting in a scar influences TST while the finding of the same TST diameter in QFT negative children with and without scar speaks against an effect of BCG scar on TST (12 mm in both groups).

Assuming that a negative QFT makes latent TB unlikely something else may have induced the large TST of ≥10 mm in many QFT negative children. This could be infections with NTM, which is unlikely because NTM would hardly give any difference between children with and without scar. Another possibility is previous TST testing with booster effect (of which we could get no information), which is also unlikely because in low-income countries with ongoing civil wars, latent TB is hardly looked for. The difference between children with and without a BCG scar makes BCG vaccination resulting in a scar the most likely possibility, even though the causes might be multifactorial. This result was unrelated to country of origin, gender and to age ≥10 years and 5–9 years. The great majority of children with a BCG scar can be assumed to have been vaccinated at birth or soon afterwards, since the World Health Organisation recommends BCG vaccination as early as possible. Since most of the children in the present study were adolescents the effect of BCG on TST seems to last for several years. This finding is in contrast with a study of school children in India stating that TST can be used for survey of TB irrespective of BCG vaccination [19] and a study from Spain indicating that BCG can give a false positive TST result in children <3 years but not in older children [20]. The most likely explanation of our finding is that BCG vaccination can induce an increase in the TST reaction, while QFT is unaffected by BCG vaccination [1–8, 20, 21].

The reasons why some children with no scar have TST ≥10 mm and negative QFT could be that they had received a vaccination without getting a scar [1, 16–18] or that the scar had waned with time which according to a study from India may occur in 30 % after a five year period [22]. Besides, different BCG vaccines have different ability to induce a scar [23, 24]. Their TST might still react with antigens in BCG. Another possibility could be that the children had been infected with NTM, many of which can induce an increase in TST without cross-reacting with the antigens in QFT [2–8]. Besides in patients with active TB negative QFT is found in about 20 % [25, 26]. In the absence of a reference diagnostic method for latent TB, it is not possible to state how common falsely negative QFT is in patients with latent TB.

The median TST diameter was significantly larger in QFT positive than in QFT negative children but the overlap was great, so it is not possible to predict the result of QFT based on the size of the TST diameter in the individual case, which agrees with two previous studies of Turkish adults and of immigrant children in Norway [27, 28].

A British study of adults, most of them from Asia, showed that chest X-ray at the port of entry contributed little or nothing to finding TB cases [12]. The present study in children agreed only partly with this. Although chest X ray findings were significantly more common in QFT positive than in QFT negative children, chest X ray in only QFT positive children would have led to missed or delayed diagnosis in two children, who had late QFT conversions. Besides there were very few children <5 years. In this age group immunologic tests like IGRA may not work well.

The study shows that TST with a cut-off value between positive and negative of 10 mm should not be used alone for the diagnosis of latent TB. This would have led to an overdiagnosis of more than two-fold in children with a BCG scar and of one-third in children without a BCG scar, in the present study. Considering the costs of prophylactic medication, side effects and controls of adherence this seems unacceptable. Confirmation with the more specific IGRA’s seems necessary both in vaccinated and nonvaccinated children. A Canadian study performed among North American Indians also concluded that TST alone is unsatisfactory for diagnosing latent TB in school children [8]. A two-step procedure with screening using the less expensive TST followed by an IGRA may be justifiable if compliance of return for reading the TST is high. A study from the United Kingdom advocates IGRA without preceding TST [12]. The U.K. study differs in two respects from the present. The immigrants in the U.K. study were mainly young adults coming from the Indian subcontinent and other Asian countries without civil wars while the patients in the present study were children and adolescents and > 50 % of them came as refugees from countries with devastating civil wars where the exposure may be expected to be high both in the home country and during the difficult journey to Europe. QFT alone is, however, not perfect for diagnosing latent or early active TB because of the late seroconversion in some patients, as shown in two cases in the present sttudy. If resources allow and if compliance for return can be expected to be good, we recommend that both TST and QFT are performed and that patients with positive TST and negative QFT are followed with repeated QFT for at least two years.

The present study can not be used for evaluation of the efficacy of BCG vaccination in neonates or early infancy but it seems obvious that any vaccine efficacy must have waned considerably. This was particularly obvious in Somalians and in unaccompanied minors. Though the children in this study were not systematically questionned about their travelling to Sweden a large number of Somalian and/or unaccompanied minors told spontaneously about several months of being hidden by smugglers in crowded vans, small apartments or tents where the risk of exposure must have been considerable. Many had also spent several months in crowded Libyan, Ethiopian, Turkish and Greek refugee camps. Children from other countries and children who came with adult relatives usually had spent less time and lived under better conditions between leaving the home countries and arrival to Sweden. Another reason for the higher proportion of Quantiferon positivity found in children coming alone or Somalian children could be that these groups had a lower frequency of BCG scars, so it is possible that the vaccination protected them during the first years of life.

## Conclusions

The study showed a significant relation between presence of BCG scar with negative QFT. It did not give a definite answer whether a BCG scar was related to size of TST. The diagnosis of latent TB in health examinations with no known exposure should not be based on a large TST alone but preferably on a combination of TST and QFT. A confirmatory IGRA should be performed in patients with large TST. QFT negative children with large TST should be repeatedly tested by IGRA. The TST diameter was larger in QFT positive than in QFT negative children but could not be used to predict the QFT result in the individual patient. Chest X ray contributes very little to the diagnosis of TB in asymptomatic QFT negative children but should not be omitted because of the possibility of falsely negative QFT or late conversion of QFT in some patients.

## Abbreviations

BCG: Bacillus calmette guérin
CRP: C-Reactive protein test
ESR: Erythrocyte sedimentation rate
IGRA: interferon gamma release assay
ns: Non-significant
NTM: Non-tuberculous mycobacteria
QFT: Quantiferon
TB: Tuberculosis
TST: Tuberculin skin test
